# A psychological intervention for engaging dialogically with auditory hallucinations (Talking With Voices): A single-site, randomised controlled feasibility trial

**DOI:** 10.1016/j.schres.2022.11.007

**Published:** 2022-12

**Authors:** Eleanor Longden, Dirk Corstens, Samantha Bowe, Melissa Pyle, Richard Emsley, Sarah Peters, Alison Branitsky, Nisha Chauhan, Nikki Dehmahdi, Wendy Jones, Natasha Holden, Amanda Larkin, Alissa Miners, Elizabeth Murphy, Ann Steele, Anthony P. Morrison

**Affiliations:** a Psychosis Research Unit, Greater Manchester Mental Health NHS Foundation Trust, Manchester, UK; b Division of Psychology and Mental Health, School of Health Sciences, Faculty of Biology, Medicine and Health, Manchester Academic Health Science Centre, The University of Manchester, Manchester, UK; c Complex Trauma and Resilience Research Unit, Greater Manchester Mental Health NHS Foundation Trust, Manchester, UK; d GGZ Noord-Holland Noord, Texel/den Helder, the Netherlands; e Department of Biostatistics and Health Informatics, Institute of Psychiatry, Psychology and Neuroscience, Kings College London, London, UK

**Keywords:** Psychotherapy, Schizophrenia, Dissociation, Treatment outcome research, Hearing Voices Movement

## Abstract

There is growing clinical interest in addressing relationship dynamics between service-users and their voices. The Talking With Voices (TwV) trial aimed to establish feasibility and acceptability of a novel dialogical intervention to reduce distress associated with voices amongst adults diagnosed with schizophrenia spectrum disorders. The single-site, single-blind (rater) randomised controlled trial recruited 50 participants who were allocated 1:1 to treatment as usual (TAU), or TAU plus up to 26 sessions of TwV therapy. Participants were assessed at baseline and again at end of treatment (six-months). The primary outcomes were quantitative and qualitative assessments of feasibility and acceptability. Secondary outcomes involved clinical measures, including targeted instruments for voice-hearing, dissociation, and emotional distress. The trial achieved 100 % of the target sample, 24 of whom were allocated to therapy and 26 to TAU. The trial had high retention (40/50 [80 %] participants at six-months) and high intervention adherence (21/24 [87.5 %] receiving ≥8 sessions). Signals of efficacy were shown in targeted measures of voice-hearing, dissociation, and perceptions of recovery. Analysis on the Positive and Negative Syndrome Scale indicated that there were no differences in means of general psychosis symptom scores in TwV compared to the control group. There were four serious adverse events in the therapy group and eight in TAU, none of which were related to study proceedings. The trial demonstrates the acceptability of the intervention and the feasibility of delivering it under controlled, randomised conditions. An adequately powered definitive trial is necessary to provide robust evidence regarding efficacy evaluation and cost-effectiveness.

Trial registration: ISRCTN 45308981.

## 1 Introduction

Hearing voices (the perception of human speech with no objective source) is considered a central feature of psychotic disorders and can lead to significant distress. However, despite robust evidence for the link between hallucinations and adversity exposure (Bailey et al., 2018; Bortolon et al., 2017; McCarthy-Jones, 2018; Varese et al., 2012), adequate access to psychological therapies is a longstanding problem within healthcare services (Colling et al., 2017; Johns et al., 2019). Given that a subset of service-users find no relief from antipsychotic medication (Harrow et al., 2014; Hassan and De Luca, 2015; Sommer et al., 2012), combined with the considerable societal and economic costs of unremittent schizophrenia (Chong et al., 2016; Jin and Mosweu, 2017), there is a clear need to refine clinical research efforts in order to expand evidence-based interventions and promote service-user choice.

In this regard, a burgeoning field of interest are therapeutic strategies which actively address the dynamics of the voice/hearer relationship. Traditionally, hallucinations were primarily viewed as a perceptual anomaly with which only limited psychological engagement was warranted. However, an emergent wave of treatments is paying greater attention to the interpersonal aspects of voice-hearing as a mechanism to promote recovery, including Relating Therapy (Hayward et al., 2017), which explores perceptions of proximity and power between hearer and voice; compassion-focused techniques (Heriot-Maitland et al., 2019), which aim to develop more empathic, accepting stances towards one's voices and oneself; Avatar (Leff et al., 2014), a dialogical strategy that employs digital representations of hostile voices, and Progressive Approach psychotherapy, which supports an attitude of empathy and acceptance from the reflective ‘Adult’ self to the distressed ‘experiencing’ self (Mosquera and Ross, 2017). The current intervention, Talking With Voices (TwV), is a form of psychotherapy which integrates such relational concepts in combination with the increasingly recognised stance that dissociative frameworks can be applied to understanding voices (including those which occur in the context of psychosis: Longden et al., 2020; Moskowitz and Corstens, 2007; Moskowitz et al., 2017). More precisely, TwV conceptualises voices as a dialogical experience which embodies different social, cultural and interpersonal factors, and is often perceived as a subjectively real event which manifests as autonomous to, and disconnected from, one's sense of self (Dorahy and Palmer, 2016; Longden et al., 2019). From this basis, it provides a format in which a therapist verbally engages with the voice(s) with the aim of facilitating a more peaceful, equitable relationship with the hearer. A full description of the therapy is provided by Longden et al. (2021), but in brief involves psychosocial education on voice-hearing, including coping strategies and recovery literature, followed by a process of psychological formulation wherein therapist and client collaborate to derive an understanding of how voices may relate to particular social/emotional conflicts and develop shared goals for the desired change in the relationship. The result of this work forms the basis for subsequent dialogue, in which the therapist poses direct queries to the voice and receives verbatim responses repeated by the client. In the long-term, the approach aims to cultivate a more constructive relationship by increasing communication and cooperation, reducing hostility, redressing unequal power dynamics, and promoting insight into voice characteristics by contextualising their associations with adverse emotions and life events. Consistent with cognitive models of psychosis (Birchwood et al., 2004; Chadwick and Birchwood, 1994; Morrison, 2001) it is also anticipated that reducing negative voice-related attributions will lead to a consequent reduction in distress.

Talking With Voices is a survivor-informed intervention (Corstens et al., 2019) and dialogical engagement with voices is an approach already utilised within the survivor-led Hearing Voices Movement (Corstens et al., 2014). However, existing clinical evidence is limited to descriptive case examples (e.g., Corstens et al., 2012; Longden and Corstens, 2020; Moskowitz and Corstens, 2007), a case series with a concurrent multiple baseline design (n = 15: Steel et al., 2019) and a small randomised controlled trial (n = 12: Schnackenberg et al., 2017). Although not all participants respond favourably to dialoguing with their voices (Steel et al., 2020), this provisional evidence indicates signals of efficacy with no emergent safety concerns. However, there is a clear need to refine such results before TwV could be considered a viable treatment option. As such, the aim of the current study was to inform the design of a definitive clinical and cost-effectiveness trial by evaluating the feasibility and acceptability of TwV therapy compared to treatment as usual (TAU) amongst adult voice-hearers with a diagnosis of a schizophrenia spectrum disorder.

## 2 Material and methods

### 2.1 Design

Talking With Voices was a single-site feasibility trial conducted according to a single-blind (rater), two-arm, randomised controlled design. Participants were recruited from Greater Manchester Mental Health (GMMH) National Health Service (NHS) Foundation Trust in northwest England, who also acted as the trial's sponsor. An additional participant was also recruited from the neighbouring Pennine Care NHS Trust. The study was funded by the National Institute of Health Research (PDF-2017-10-05) and prospectively registered with the ISRCTN (45308981). Ethical approvals were received from the North West–Preston Research Ethics Committee (17/NW/0633).

### 2.2 Participants

Eligible participants were aged ≥18 years; had heard voices for at least one year and scored ≥4 on the auditory hallucination subscale of the Positive and Negative Syndrome Scale (PANSS: Kay et al., 1987); had no medication changes within the past month; met criteria for ICD schizophrenia spectrum disorder; were able to provide written, informed consent; were not currently receiving structured psychological therapy for psychosis; were in contact with secondary care mental health services and had a care coordinator; were willing and able to communicate with their voices and relay voice utterances to a therapist; and heard voices which were sufficiently personified to engage in dialogical work.

Individuals meeting any of the following criteria were subsequently excluded: at immediate risk of harm to self or others, non-English speaking, in receipt of a primary diagnosis of alcohol/substance dependence or autism spectrum disorder, demonstrating a moderate/severe learning disability, having an organic brain injury or illness implicated in psychotic symptoms, scoring >5 on the conceptual disorganization subscale of the PANSS, or being homeless and/or of no fixed abode.

Participants were primarily referred to the trial by staff within community mental health teams (CMHTs) or early intervention (EI) services across the host site. Eligibility data were derived from administering standardised measures and through liaison with participants and relevant healthcare workers.

### 2.3 Randomisation

Participants were randomly allocated in a 1:1 ratio to receive either TAU alone, or TAU plus therapy. Randomisation to conditions was conducted by the trial administrator via the secure online service SealedEnvelope.com and was independent and concealed with outcome assessors blinded to treatment group. It was not stratified for any variables and employed randomised-permuted blocks of 4, 6 and 8. Allocation was made known to the chief investigator (EL) to monitor adherence to the randomisation algorithm, the trial therapists, the trial administrator, and communicated to participants and their healthcare teams via phone call and letter. A standard operating procedure for allocation concealment, including an adherence declaration, was read and signed by trial staff and the importance of blinding was impressed upon participants, their family members, and healthcare workers. Any breaks were reported to the chief investigator, with learning points/remedial action disseminated within the research team, then submitted to the combined independent Trial Steering and Data Monitoring and Ethics Committee (TSC-DMEC) for oversight purposes.

### 2.4 Procedure

Participants were allocated to receive either treatment as usual (TAU), or up to 26 one-hour sessions of TwV therapy plus TAU over six months. The intervention was delivered by trained clinical psychologists, with sessions typically offered once a week according to a structured protocol (Longden et al., 2021). The initial phase focused on engagement, psychosocial education, and development of coping/self-soothing strategies, followed by assessment and formulation of voices, dialogical work, and a final period of evaluation and outcome consolidation. Electronic session records and adherence checklists were utilised to maximize fidelity, with any protocol divergences monitored during therapist supervision. These sessions occurred fortnightly using a group peer-supervision format and were facilitated by a psychiatrist with clinical experience of TwV (DC) and two researchers with lived experience of voice-hearing (EL & AB). Unless requested otherwise, therapy was conducted in participants' homes in a manner consistent with assertive outreach practice, with participants seen by the same therapist whenever possible to maintain consistency and engagement. In the UK, TAU for service-users with psychosis is based on the Care Programme Approach and can comprise a range of interventions, including psychiatric medication, care coordination, rehabilitative and family intervention services, outpatient follow-up care, and access to cognitive behavioural therapy for psychosis (CBTp).

### 2.5 Outcomes

The primary outcomes were evidence for the feasibility and acceptability of delivering the intervention under randomised conditions, including quality of data collection, retention and recruitment rates, adherence to allocation, and treatment acceptability (assessed through discontinuation rates and a nested qualitative study). A three-stage model for progression criteria was utilised to establish the viability of progressing to a definitive trial, which was approved in advance by the TSC-DMEC and related to baseline recruitment, retention at six-month follow-up, and adherence to therapy. Specific criteria for progression were at least 80 % of the target population (green zone), 60–79 % (amber zone) or <59 % (red zone), and were operationalised using randomisation rates, completion of the PANSS at six-month follow-up, and attendance of at least eight therapy sessions.

A number of secondary outcomes were also chosen to help identify relevant clinical variables and potential mechanisms of action for the intervention, as well as to assess the applicability and acceptability of collecting these in the event of a definitive trial. These included specific measures of voice-hearing (the Voice and You scale [VAY: Hayward et al., 2008], the Subtypes of Voice Hearing Questionnaire [Cox et al., 2017], the Revised Beliefs about Voices Questionnaire [BAVQ-R: Chadwick et al., 2000], and the PANSS hallucinations subscale) and general clinical presentation (the Questionnaire About the Process of Recovery [QPR: Neil et al., 2009], the revised Dissociative Experiences Scale [DES-II: Carlson and Putnam, 1993], and PANSS subscales). Additional assessments included adversity exposure (the Revised Life Stressor Checklist [LSC-R: Wolfe et al., 1996]) and health economics data (the EQ-5D: van Reenen and Janssen, 2015). Measures were administered at baseline and six-month follow-up by trained researchers who were blind and independent to treatment group. Participants in the intervention arm additionally completed a measure of therapeutic alliance, the Working Alliance Inventory (Tracey and Kokotovic, 1989), and a customised therapy evaluation, both dispensed by trial therapists. A nested qualitative evaluation was also conducted amongst trial therapists (Longden et al., 2022) and participants (Longden et al., in preparation).

Serious adverse events (SAEs) and adverse events (AEs) were recorded via participant self-report to therapists and/or research assistants during the trial. Screening of electronic medical records was also conducted at follow-up by a researcher who was not masked to allocation. All SAEs were reported to the TSC-DMEC for independent monitoring.

### 2.6 Statistical analysis

A target sample of 50 participants was deemed sufficient to both demonstrate feasibility and to obtain reliable parameter estimates for sample size in a definitive trial (Browne, 1995). Given that the focus of analysis was not hypothesis testing, a formal power calculation for detecting treatment differences was not conducted. Instead, a focus was placed on descriptive statistics, point estimates, and associated 95 % confidence intervals rather than tests of statistical significance. Descriptive baseline and follow-up data were summarised as mean (SD) for continuous variables and frequencies/percentages for categorical variables. Analyses followed a pre-specified plan approved by the chief investigator, the trial statistician, and the TSC-DMEC (available to view online at www.isrctn.com/ISRCTN45308981) and was based on intention-to-treat principles at the participant level. Linear regression was used to estimate the between-group adjusted mean difference controlling for baseline scores. All available data was used from each timepoint, with missing data imputed with pro-rating. The main analyses were all conducted in Stata (v.16: StataCorp, 2019).

## 3 Results

**Fig. 1:**
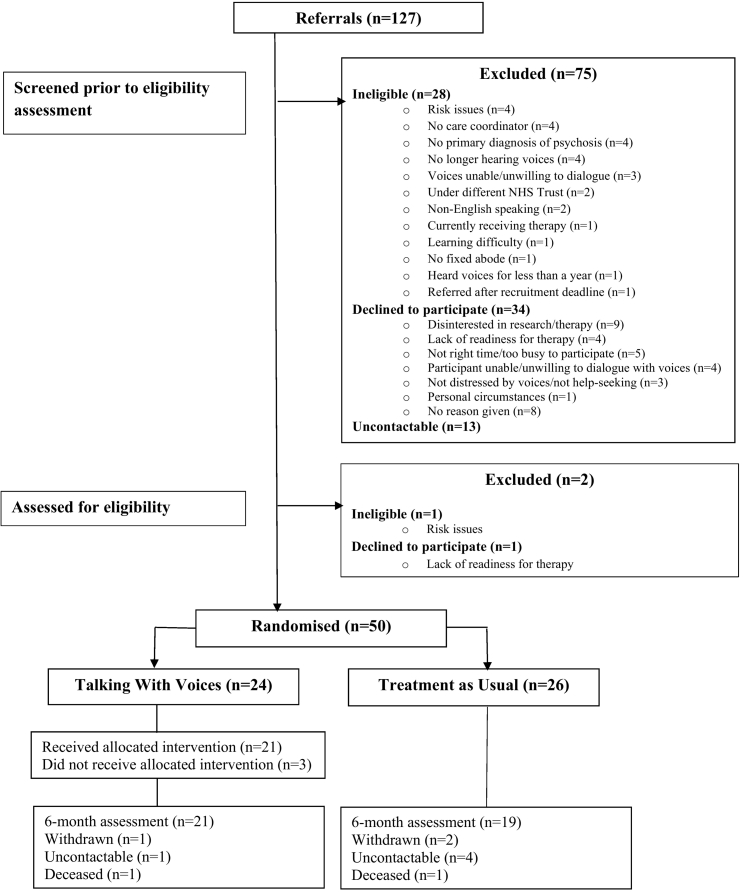
CONSORT diagram for flow of participants.

A total of 127 individuals were referred to the trial between 08 February 2018 and 16 December 2019; 50 of whom were recruited for the study, with 24 allocated to TwV and 26 to TAU. Follow-up assessments were conducted between 12 September 2018 and 05 August 2020. Baseline characteristics are summarised in Table 1 and a CONSORT diagram depicting participant flow is presented in Fig. 1.

**Table 1:** Baseline characteristics.

	Total sample (N = 50)	Therapy (N = 24)	TAU (N = 26)
	Mean (SD) or N (%)	Mean (SD) or N (%)	Mean (SD) or N (%)
Age	39.56 (11.55)	38.1 (10.1)	40.9 (12.8)
Sex			
Female	23 (46)	14 (58)	9 (35)
Male	27 (54)	10 (42)	17 (65)
Ethnicity			
White background	33 (66)	12 (50)	21 (81)
Asian	7 (14)	6 (25)	1 (4)
Black	3 (6)	2 (8)	1 (4)
Mixed background	4 (8)	2 (8)	2 (8)
Other ethnic group	3 (6)	2 (8)	1 (4)
Employment status			
Unemployed	39 (78)	18 (75)	21 (81)
Employed full-time	2 (4)	1 (4)	1 (4)
Employed part-time	4 (8)	3 (13)	1 (4)
Retired	2 (4)	0 (0)	2 (8)
Student	2 (4)	1 (4)	1 (4)
Voluntary	1 (2)	1 (4)	0
Highest educational level			
Primary	3 (6)	2 (8)	1 (4)
Secondary	20 (40)	8 (33)	12 (46)
Further/college	12 (24)	6 (25)	6 (23)
Higher/university	13 (26)	8 (33)	5 (19)
Did not respond	2 (4)	0	2 (8)
Diagnosis			
Schizophrenia	33 (66)	16 (67)	17 (65)
Schizoaffective	2 (4)	1 (4)	1 (4)
Psychosis (other)	15 (30)	7 (29)	8 (31)
Current antipsychotic medication			
Yes	48 (96)	24 (100)	24 (92)
No	2 (4)	0	2 (8)
Previous therapy for voices			
Yes	20 (40)	7 (29)	13 (50)
No	27 (54)	14 (58)	13 (50)
Did not respond	3 (6)	3 (13)	0
Voice-hearing duration (years)	11.90 (11.04)	10.8 (8.4)	13.0 (13.2)

In terms of feasibility criteria, recruitments rates were 100 % of the target sample (green progression zone). The conversion rate was around 2:1, with 35 (27.6 %) of the 127 referred individuals declining to participate and an additional 29 (22.8 %) being ineligible. The most frequently referring services were CMHTs, responsible for the referrals of 36 (72 %) participants.

Forty participants were retained in the trial at the six-month (end of treatment) assessment (80 %; green). Attrition was low, with seven participants being uncontactable and three formally withdrawing from the trial. Of the latter, one was from the therapy group owing to a reluctance to engage with the intervention and two were from TAU, one of whom cited disappointment with his allocation and the second who did not provide a reason. The number of full blind breaks was four, three of which occurred in the therapy group, and all of which resulted from participants disclosing their allocation to research workers. Two of these breaks took place prior to the six-month assessment and were transferred to a new and independent assessor. The remaining two occurred during the assessment process, in which case audio recorded PANSS interviews were independently rated by a separate assessor.

Of the 24 participants in the treatment group, 21 (87.5 %) received at least eight therapy sessions (green), the pre-defined feasibility criteria for adherent delivery of TwV. The mean number of attended sessions was 17.42 (SD = 8.3; range 2–39). Three individuals did not receive their allocated intervention, owing to the death of the participant by suicide after two sessions, withdrawal from the study after two sessions, and disengagement from therapy after six sessions. Adherence to the trial's therapy manual was high with key milestones met by a majority of participants. Specifically, of participants receiving at least eight sessions, 21 (100 %) had a psychological formulation developed; 18 (85.7 %) undertook at least one voice session of dialogue work; and targeted techniques to facilitate an improved relationship between client and voice(s), ‘time-sharing’ and ‘developing short replies’, were achieved by 14 (66.7 %) and 17 (80.9 %) respectively.

In our intention-to-treat analysis of clinical outcomes, pro-rated scores for the secondary measures at 26 weeks were assessed for all participants still retained by this endpoint. Descriptive statistics, point estimates of the adjusted mean difference, standard errors and associated 95 % confidence are presented in Table 2 for each randomised group.

**Table 2:** Baseline and follow-up scores for both groups on the secondary assessment measures.

	TwV(N = 24)	TAU (N = 26)	Adjusted mean difference (SE)	95 % CI
	M(SD); n	M(SD); n		
PANSS total				
Baseline	67.5 (13.5); 24	63.2 (9.0); 26		
26 weeks	59.7 (15.6); 21	56.3 (15.3); 19	2.79 (3.80)	−4.91, 10.49
PANSS positive				
Baseline	18.8 (4.9); 24	17.6 (3.5); 24		
26 weeks	17.1 (5.2); 21	15.7 (5.0); 19	0.40 (1.35)	−2.34, 3.14
PANSS negative				
Baseline	13.3 (4.0); 24	12.8 (4.2); 26		
26 weeks	12.0 (4.2); 21	11.7 (5.9); 19	0.44 (1.32)	−2.23, 3.11
PANSS general				
Baseline	35.5 (7.2); 24	32.8 (5.3); 26		
26 weeks	30.5 (8.1); 21	28.9 (7.4); 19	1.29 (1.90)	−2.56, 5.14
PANSS anxiety item				
Baseline	4.4 (1.2); 24	4.3 (1.2); 26		
26 weeks	3.7 (1.4); 21	3.3 (1.6); 19	−0.41 (0.43)	−1.29, 0.46
PANSS depression item				
Baseline	4.4 (1.0); 24	3.8 (1.3); 26		
26 weeks	3.4 (1.6); 21	3.5 (1.4); 19	0.39 (0.43)	−0.49, 1.26
PANSS hallucinations item				
Baseline	5.2 (0.6); 24	5.2 (0.6); 26		
26 weeks	4.7 (1.2); 21	4.0 (1.8); 19	−0.61 (0.47)	−1.57, 0.34
DES-II				
Baseline	30.9 (20.4); 20	28.1 (20.9); 20		
26 weeks	25.8 (25.5); 14	28.6 (23.6); 16	7.22 (7.17)	−7.65, 22.08
QPR				
Baseline	32.3 (10.8); 22	31.9 (11.9); 23		
26 weeks	38.5 (12.2); 16	30.8 (15.9); 16	−6.94 (4.41)	−16.00, 2.12
VAY dominance				
Baseline	13.8 (6.0); 20	12.7 (5.7); 23		
26 weeks	10.9 (6.0); 15	13.4 (6.8); 16	2.72 (1.85)	−1.08, 6.53
VAY intrusiveness				
Baseline	10.2 (4.0); 19	8.0 (3.7); 22		
26 weeks	8.8 (3.6); 15	10.3 (4.3); 15	1.49 (1.30)	−1.20, 4.17
VAY dependence				
Baseline	9.4 (6.8); 20	7.3 (5.3); 21		
26 weeks	10.3 (8.1); 13	9.0 (8.0); 16	1.36 (2.57)	−3.98, 6.69
VAY distance				
Baseline	12.9 (4.8); 18	11.2 (5.3); 22		
26 weeks	11.5 (5.2); 14	12.3 (6.1); 16	1.63 (1.55)	−1.59, 4.86
BAVQ-R omnipotence				
Baseline	12.0 (4.2); 21	10.8 (3.9); 23		
26 weeks	11.5 (4.6); 16	9.4 (2.9); 16	−0.94 (1.38)	−3.78, 1.90
BAVQ-R malevolence				
Baseline	11.1 (5.3); 21	8.6 (4.4); 23		
26 weeks	9.3 (6.2); 16	8.3 (5.1); 16	2.03 (1.58)	−1.21, 5.27
BAVQ-R benevolence				
Baseline	4.1 (4.3); 21	5.8 (5.6); 23		
26 weeks	6.2 (5.3); 16	4.5 (6.2); 16	−3.93 (1.63)	−7.27, −0.58
BAVQ-R emotional resistance				
Baseline	8.4 (3.3); 21	7.3 (2.9); 22		
26 weeks	6.9 (4.5); 16	7.3 (3.1); 16	2.00 (1.09)	−0.25, 4.24
BAVQ-R behavioural resistance				
Baseline	10.0 (4.3); 21	10.4 (4.9); 23		
26 weeks	8.8 (4.4); 16	9.9 (4.4); 15	0.89 (1.30)	−1.78, 3.56
BAVQ-R emotional engagement				
Baseline	3.0 (3.1); 21	3.4 (3.7); 22		
26 weeks	5.6 (4.4); 16	2.8 (4.4); 16	−3.07 (1.40)	−5.94, −0.20
BAVQ-R behavioural engagement				
Baseline	3.3 (2.3); 21	3.5 (2.9); 21		
26 weeks	4.6 (3.3); 16	3.1 (3.8); 15	−1.62 (1.10)	−3.88, 0.64

The safety assessment is summarised in Table 3 and indicated that twice as many participants experienced SAEs in the TAU arm than those receiving therapy, most notably voluntary admissions to psychiatric hospital. No SAEs in either group were deemed related to trial procedures. Other non-serious AEs were experienced in comparable rates across groups, the majority of which were incidents of self-injury.

**Table 3:** Incidence of adverse events across groups.

	Therapy N = 24	TAU N = 26
Serious Adverse Events		
Participants with an SAE	4 (16.7 %)	8 (30.8 %)
Number of SAEs	5	13
Types of SAE		
Death	1	1
Voluntary psychiatric admission	1	6
Involuntary psychiatric admission	2	3
Otherwise medically significant (overdose)	1	2
Otherwise medically significant (aspiration during ECT)	0	1
Adverse Events		
Participants with an AE	5 (20.8 %)	7 (26.9 %)
Number of AEs	14	16
Types of AE		
Self-injury	9	9
Suicidal ideation with behavioural component	2	3
Seizure/loss of consciousness	1	1
A&E attendance	1	1
Deterioration in mental state	1	2

## 4 Discussion

The TwV trial shows that it is possible to recruit, retain and engage service-users who meet criteria for schizophrenia spectrum disorders to evaluate a dialogical intervention for auditory hallucinations under randomised controlled conditions, thus providing indications of both feasibility and acceptability. This is a promising development, given the growing evidence which suggests that auditory hallucinations, at least for some individuals, are psychologically meaningful events in which demonstrable links exist between adversity exposure and the responses to, and content of, the voices people hear. However, while addressing the interpersonal and relational dynamics of voice-hearing may be an encouraging treatment strategy, more definitive evidence is needed before such interventions can be routinely offered within healthcare services.

The trial had low attrition (20 % at six months) and therapy delivery was highly satisfactory, with a sizeable proportion of participants receiving their allocated intervention. Although withdrawal rates can be in the approximate region of 18 % for therapies which include some element of aversive exposure, including trauma-focused treatments (Imel et al., 2013) and direct work with voices (Craig et al., 2016), only three participants (12.5 %) attended less than eight sessions of TwV, only one of whom withdrew from the trial (4.2 %). Participants receiving therapy additionally had fewer SAEs than the control arm, none of which were considered related to the treatment or trial proceedings. These findings must be considered in the context of the small sample, yet suggest that direct therapeutic engagement with voices, including their association with adverse life events, may be a safe and acceptable treatment option for many service-users with psychosis. In this regard, the sample appeared broadly generalizable to community-based populations in terms of elevated rates of positive symptoms, high medication usage and low rates of employment, although an additional finding of note may be that a third of participants did not identify as White. This is a higher proportion than CBTp trials recruiting from the same geographic region (8.6 %–14.2 %: Law et al., 2017; Morrison et al., 2018; Morrison et al., 2020; Morrison et al., 2013) and, if replicated in a larger sample, may have implications for diversity and inclusion in that an intervention premised on relational strategies may appeal to such service-users; possibly because they are more likely to perceive their experiences as having a spiritual/cultural origin (Singh et al., 2015) and may, therefore, be particularly receptive to dialogical engagement.

A definitive randomised controlled trial is now required to determine the clinical and cost-effectiveness of the TwV intervention. Although the study was not powered to detect treatment effects, the results suggest TwV may have beneficial outcomes for how voices are perceived (particularly in terms of increased benevolence), as well as facilitating recovery and reducing dissociation. Additional research is now necessary to investigate potential treatment mechanisms that may explain these effects, as well as how they relate to more established clinical and functional outcomes such as quality of life and other psychiatric symptoms. Future research could also help facilitate preference and informed choice for therapies, dependent on the personal treatment goals of an individual service-user, by examining the relative effectiveness and differential impact of TwV on different treatment targets (e.g., relationships with voices, voice-related distress, voice frequency and duration, and quality of life and functioning) compared with other evidence-based psychological interventions (e.g., CBTp, Avatar therapy). In this regard, future research could also seek to identify who TwV is most suitable for (including whether certain subtypes of voices respond better [Smailes et al., 2015], and whether there are any subgroups of client for whom it may be contra-indicated).

There are a number of design considerations for a future definitive trial that have been identified by our feasibility study. Although resource limitations prohibited more than two assessments, a definitive trial should include a longer-term follow-up in order to evaluate the longevity of any treatment effects. Secondly, flexibility regarding the timing and venue for assessment/therapy appointments proved crucial for engagement, meaning sufficient staffing capacity should be planned to facilitate this in the future. In terms of therapy delivery, one participant commented on feeling unprepared for TwV's emphasis on adverse life events, whereas a second stated strong opposition to its premise of improving the relationship with one's voices. These elements should receive a greater emphasis prior to consent, with both verbal briefings and written materials being clear about the presence and purpose of discussions regarding traumatic events and that the therapy does not explicitly aim for voice cessation. An additional participant also revealed post-randomisation that he had entered the trial to educate professionals about his personal theory of hallucinations rather than to receive the intervention. Adding ‘help-seeking’ as an inclusion criterion may reduce the likelihood of recruiting those who are unmotivated for therapy, with more focussed screening by assessors additionally helping to identify whether participants intend to engage with TwV and understand its premise. It was further noted that participants required substantial time and appointment flexibility to engage in, and feel safe with, the therapeutic process. It may therefore be a worthwhile amendment to extend the therapy window from 6 to 9 months (and/or incorporate periodic booster sessions) to facilitate the consolidation and reinforcement of therapeutic gains. In turn, while it is striking that most participants were referred from CMHTs relative to other services we do not have data to explain why this occurred. Speculatively, it may reflect low access to psychological therapies in community teams, or possibly from participants' voice characteristics and their resulting beliefs and responses to them; both of which may have motivated referrals, either separately or in combination. A future trial could capture these factors more precisely. Final considerations for a definitive trial also regard the choice of outcome measurement. The PANSS did not adequately capture the intended voice-related targets of the intervention (namely relationship with, and impact of, the voices) and a more sensitive scale for assessing voice severity (e.g., the PSYRATS: Haddock et al., 1999) should be employed.

## 5 Conclusions

As an under-powered feasibility study the clinical implications of this research are limited, and a sufficiently resourced effectiveness trial will now be required to obtain more definitive evidence of TwV's clinical impact. However, it is apparent that clinicians can engage in therapeutic dialogues with voices and that such voices, at least for some service-users, can be conceptualised, engaged with, and ameliorated within the context of individual life histories. Based on this study, it is reasonable to propose that relational dynamics can be an important component of the voice-hearing experience and, beyond actual dialogue, should still be considered when supporting clients who are distressed by the voices they hear.

## Role of the funding source

The study was funded by a 10.13039/501100000272National Institute for Health Research (NIHR) Postdoctoral Fellowship Scheme for EL (PDF-2017-10-050). RE is part funded by the NIHR 10.13039/100014461Biomedical Research Centre at 10.13039/100009362South London and Maudsley NHS Foundation Trust and 10.13039/100009360King's College London and is supported by an NIHR Research Professorship (NIHR300051). The funders had no involvement in either the conduct of the research or the preparation of the article.

## Declaration of competing interest

Three authors (EL, DC and AB) have received financial payments for delivering teaching or supervision for the Talking With Voices approach. There are no other reported conflicts of interest.
